# Using intervention mapping to develop and adapt a secondary stroke prevention program in Veterans Health Administration medical centers

**DOI:** 10.1186/1748-5908-5-97

**Published:** 2010-12-15

**Authors:** Arlene A Schmid, Jane Andersen, Thomas Kent, Linda S Williams, Teresa M Damush

**Affiliations:** 1 Roudebush Veterans Administration Medical Center; Health Services Research and Development (HSR&D) Center on Implementing Evidence-Based Practice, 1481 W. 10th Street, 11 H, Indianapolis, Indiana 46202-5199, USA; 2 VA Stroke Quality Enhancement Research Initiative (QUERI), 1481 W. 10th Street, 11 H, Indianapolis, Indiana 46202-5199, USA; 3 Indiana University School of Health and Rehabilitation Science, Department of Occupational Therapy, 1140 W. Michigan Street CF 311, Indianapolis, Indiana 46202-5199, USA; 4 Indiana University Center for Aging Research 1001 West 10th Street, Indianapolis, Indiana 46202-5199, USA; 5 Michael E. DeBakey Veterans Administration Medical Center, 2002 Holcombe BlvdHouston, TX, USA; 6 Regenstrief Institute, 1001 West 10th Street, Indianapolis, Indiana 46202-5199, USA; 7 Indiana University School of Medicine, Department of Neurology, 1001 West 10th Street, Indianapolis, Indiana 46202-5199, USA; 8 Indiana University School of Medicine, Department of General Internal and Geriatrics, 1001 West 10th Street, Indianapolis, Indiana 46202-5199, USA

## Abstract

Secondary stroke prevention is championed by the stroke guidelines; however, it is rarely systematically delivered. We sought to develop a locally tailored, evidence-based secondary stroke prevention program. The purpose of this paper was to apply intervention mapping (IM) to develop our locally tailored stroke prevention program and implementation plan. We completed a needs assessment and the five Steps of IM. The needs assessment included semi-structured interviews of 45 providers; 26 in Indianapolis and 19 in Houston. We queried frontline clinical providers of stroke care using structured interviews on the following topics: current provider practices in secondary stroke risk factor management; barriers and needs to support risk factor management; and suggestions on how to enhance secondary stroke risk factor management throughout the continuum of care. We then describe how we incorporated each of the five Steps of IM to develop locally tailored programs at two sites that will be evaluated through surveys for patient outcomes, and medical records chart abstraction for processes of care.

## Background

The development of an implementation intervention is complex and involves many components. Often the outcomes of such interventions are published without the details of how the intervention was developed or from where the components were derived [1]. Intervention mapping (IM) is a technique used to develop an evidence-based intervention that provides and balances both theoretical and practical strategies while incorporating formative evaluation, a needs assessment, program development, and evaluation [2]. We used IM to guide us through the development of a theory-based, multi-site, secondary stroke prevention program.

### Stroke prevention

The used of an evidence-based intervention to manage stroke risk factors could have great impact due to the high prevalence of stroke, with approximately 795,000 people in the United States sustaining a stroke annually [3]. With its deleterious effects, stroke is classified as the most disabling chronic disease with negative consequences for individuals, families, and society [4,5]. Future stroke risk increases after a cerebrovascular event [6]; importantly, 200,000 of all strokes are recurrent strokes. For example, more than 12% of those with stroke or transient ischemic attack (TIA) experience a second stroke within the year [7,8]. This increased risk persists for at least five years [9]. Furthermore, 15% of strokes are preceded by a TIA [10]. Significantly, the risk of death is doubled after a second stroke [11].

Such a cerebrovascular event may be an opportunity for targeting secondary stroke prevention [12]. Hoenig and colleagues reported that stroke survivors often continue unhealthy lifestyle choices regarding stroke risk factors and are therefore at increased risk for a second stroke [13]. Despite knowledge and impact of risk reduction, clinical providers may not aggressively counsel or treat patients with behavioral or medical interventions for stroke prevention [14].

Prevention of a first or second stroke is possible by identifying and controlling stroke risk factors [15]. While some risk factors are permanent (*e.g*., age, hereditary), the majority are modifiable (*e.g*., atrial fibrillation, obesity, tobacco and alcohol use, hypertension, and physical inactivity) [16,17]. Modifiable risk factors are best managed through lifestyle and medication management. To achieve optimal management, it is likely that providers and stroke survivors will need to work together through complex interventions to truly prevent a secondary stroke [18-20].

Clinical and practice guidelines are common and exist for post-stroke care. Such guidelines are developed to guide practice and generally consist of a guideline text, a one-page summary, and a significant background document including recommendations based on levels of evidence. Stroke care guidelines, such as the Veterans Administration/Department of Defense (VA/DoD) Stroke Rehabilitation Guidelines, the Agency for Healthcare Research and Quality (AHRQ) Clinical Guidelines For Stroke, and the American Stroke Association all advocate for the implementation of secondary prevention programming that addresses stroke risk factor modification after a cerebrovascular event [7,21-23]. Although there are resources for the management of some risk factors -- *e.g*., blood pressure (BP) and diabetes -- these resources are not routinely targeted to or used by veterans with recent stroke or TIA. We are not aware of any systematic programming or standardized support available in the VA to enhance stroke risk factor management. Thus we have used IM to guide us in the planning, development, and implementation of a complex stroke prevention program.

### Intervention mapping

Given the effect of stroke on morbidity and health-related quality of life, interventions designed to address the needs of stroke survivors and their providers are complex and involve multilevel strategies to produce system and individual changes to improve outcomes. Planning for the implementation of such complex interventions may be guided through IM [2,24]. IM is a process for developing theory and evidence-based programs, and is used to provide a systematic framework for planning, development, and implementation of health promotion and prevention programs [2,24-34]. For example, IM has been used in guiding program development and implementation for adapting effective sexually transmitted disease and pregnancy programs [33], for applying health psychology theory to prevention programs [34], in designing an occupational health guideline to prevent weight gain among employees [26], and other health promotion and prevention programs. IM helps the user to apply a framework or a model by operationalizing the theoretical components to link performance objectives with intervention methods and implementation strategies [2,24,28]. The result of IM is a systematic and practice-friendly process for implementing evidence-based programming [33].

## Methods

We employed IM techniques, including a needs assessment, to develop a systematic stroke prevention program locally tailored to two healthcare facilities within a national organization. This was completed to support a VA Health Services Research and Development Implementation grant: Teaching Others tOLive with Stroke (TOOLS). TOOLS focuses on implementing existing stroke prevention tools into usual care at two VA medical centers (VAMCs). All research reported in this study was approved by both sites' local institutional review boards and VA research and development committees.

### Intervention Mapping

Bartholomew and colleagues identified the five Steps of IM [2]. The Steps and subsequent tasks of IM include a planned process using matrices for the systematic development, implementation, and evaluation of the program. In addition to a needs assessment (Step 0), IM includes the following five Steps (See Table 1 for Steps and tasks): 1) creation of a matrix of proximal program objectives; 2) selection of theory-based intervention methodologies (the Chronic Care Model [35] was used to organize the elements of the healthcare system, practice delivery, and patient self-management, and the Theory of Planned Behavior [36] was used to guide the implementation strategies) practical strategies and suggestions from targeted users; 3) design and organization of the program; 4) adoption and implementation of the program; and 5) monitoring and program evaluation [2]. We completed a needs assessment and utilized the five Steps of IM to develop our intervention program and implementation strategies, and report the results.

**Table 1:** Steps of Intervention Mapping (IM) 2

	Step	Tasks
0	Needs assessment	Specify needs of providers
		Specify needs of patients

1	Creation of a matrix of proximal program objectives	Specify the performance objectives
		Specify important, changeable determinants
		Differentiate the target population
		Create matrices of proximal program objectives

2	Selection of theory based intervention methodologies practical strategies and suggestions from targeted users	Brainstorm methods to achieve proximal program objectives
		Use the theoretical and empirical literature to further delineate the methods
		Translate methods into strategies

3	Design and organization of the program	Operationalize the strategies into plans considering implementers and sites
		Design instruction materials
		Pretest instruction materials with the target group
		Produce the materials

4	Adoption and implementation of the program	Develop a linkage system
		Specify adoption and implementation performance objectives
		Specify determinants of adoption and implementation
		Write and implementation plan

5	Monitoring and program evaluation	Develop an evaluation model using information from the previous Steps of IM and information from the needs assessment
		Develop effect evaluation questions, referring to the matrices of proximal program objectives as blueprints for instrument development
		Develop process evaluation questions from the needs assessment and intervention map

#### Step 0: Needs assessment

In order to develop an intervention program to locally tailor and implement the use of available tools for secondary stroke prevention into an existing healthcare system, we began with a needs assessment of the targeted users of the program. We conducted the needs assessment using semi-structured interviews to elicit providers' needs and barriers to systematic delivery of secondary stroke prevention, and preferences and suggestions for program elements and implementation strategies to guide our IM and future implementation program [28,29]. Because our planned intervention targeted both providers of stroke care and stroke patients, we also conducted focus groups with key stakeholders, the veteran stroke survivors, and their caregivers to understand their barriers to and preferences for secondary stroke prevention services. Those results are published elsewhere and incorporated into the patient self-management element of the program [37].

We based our semi-structured interviews on elements of the chronic care model [38], the components of guideline care for secondary stroke prevention [39], and practical strategies currently used. For example, we included questions from the decision support domain of the chronic care model that queried providers on the use of health services tools (for example, computer reminders and use of pocket cards). For guideline care, we included the components delineated by the VA/DoD and the American Stroke Association: ordering tests, prescribing medication, assessing and counseling on risk factors, and making referrals to local community resources and programs.

Specifically, this aspect of the TOOLS study focused on multiple providers who represented the continuum of stroke care at the Indianapolis and Houston VAMCs: neurologists; neurology residents; general internists; physician assistants; nurse practitioners; nurses; occupational, physical, and recreational therapists; and social workers. We conducted all interviews in a one-on-one setting. We evaluated their current roles/perceived roles in secondary stroke prevention and the current state of and capacity for stroke prevention programming. We also sought to gain their guidance as we moved forward to develop, implement, and evaluate the TOOLS program. Specifically, the objectives of the needs assessment were to: determine provider perceptions of their current role and practices in secondary stroke prevention; identify the needs to support providers in providing secondary stroke prevention; and elicit practical suggestions for improving the delivery of secondary stroke prevention at the local site (Table 2). These semi-structured interviews were synthesized and used to plan our local adaptation of the secondary stroke prevention program and evaluation.

**Table 2:** Summarization of the recommendations and next actions for the TOOLS intervention

Enhance provider practices in secondary stroke risk factor management	Address the needs to support providers in secondary stroke risk factor management	Implement advice from providers to enhance secondary stroke risk factor management throughout the continuum of care
Educate all types of providers regarding stroke warning signs, stroke risk factors, and stroke risk factor management	Tailor the self-management aspect of the TOOLS intervention to each veteran using self-management concepts	Address secondary stroke prevention prior to discharge - we are providing this through training of all providers
Teach rehabilitation therapists to include a stroke risk factor management goal for every patient with stroke or TIA	Develop and issue rehabilitation specific information handouts and pamphlets for addressing stroke risk factors	Send pamphlets and information home with each patient - we are addressing this through nursing discharge
Incorporate (through nursing) secondary stroke risk factor management information and training into the discharge process for every patient with stroke or TIA	Develop and issue a self-management prescription pad for risk factors - this will provide information for clinics, etc	Need to establish a gatekeeper (or champion) at each facility, we feel that this person may be found in rehabilitation due to the relationships that are often built
		Develop a discharge template
		Initiate peer to peer programming and facility stroke support groups

### Interview

We developed semi-structured interview guides that were based on the chronic care model with questions from the model domains including: the local community resources available and utilized; patient self-management; delivery system at discharge and follow up care; decision support during hospitalization; and discharge and follow up visits [38]. A team of healthcare providers and researchers first reviewed and critiqued the interview questions. We then pilot tested the interview questions with four providers and made modifications based on their recommendations.

We included probes throughout the interviews to delve into the research topics: current knowledge and practices to prevent a second stroke; needs to support providers in providing secondary stroke prevention to secondary stroke prevention; and resources necessary to provide enhanced secondary stroke prevention. In addition, the interviews were specific to disciplines and the role responsibilities of each provider type. For example, rehabilitation therapists were not asked about prescribing medications to manage BP. A sample interview guide is available from the authors upon request.

The interviews were completed in both Houston and in Indianapolis by four experienced research staff trained by the investigator (TD) on interviewing techniques, including how to probe based upon given responses. The interviewers practiced administering the interview on study staff. In total, there were 26 completed interviews in Indianapolis and 18 in Houston. All interviews were audiotaped and transcribed into word processing files for data analysis. All provider identifiers were removed.

### Findings of needs assessment

We interviewed 44 providers; 26 in Indianapolis and 18 in Houston (Table 3). Most importantly, almost all providers endorsed the idea that they have a role in secondary stroke risk factor management (81% in Indianapolis and 100% in Houston). However, there was a disparity in the extent and delivery manner of this role. Some consistent themes that emerged from our needs assessment that guided our IM included a need for: improved patient and caregiver compliance; standardized clinical reminders or prevention checklist; training regarding stroke risk factors and warning signs; stroke support groups; and provision of pamphlets and written information. These topics and emergent themes were used to support IM Steps and are described below.

**Table 3:** Type and location of provider interviews and indication of the number of providers (by type) that commented on each theme, n = 44.

Provider type	n	Current provider practices in secondary stroke risk factor management			Barriers and supports to risk factor management						Advice or needs to enhance secondary stroke prevention					
		**The provider is providing secondary stroke prevention**	**Works with other providers/** **referrals**	**Works with pt, family, caregiver**	**Adherence and motivation**	**Provider lacks knowledge**	**Lack of admin support**	Other*	**Pt cognition/education**	**Transport-ation**	**Wants education**	**Wants handouts**	**Wants check off list**	**Wants support groups**	**How to refer to what?**	**Other^**

**Indianapolis, IN**																
MD	2	2	1	1	1	2	0	2	0	0	2	1	0	1	1	2
Res	3	3	0	1	1	0	3	2	3	0	2	3	1	0	3	2
RN	4	4	1	1	4	0	2	1	3	0	1	2	0	0	0	1
OT	5	5	5	0	5	0	3	2	1	0	5	5	1	1	1	1
PT	4	4	4	0	4	0	3	3	2	0	3	3	1	0	2	3
RT	2	2	2	0	2	0	2	0	0	0	1	2	0	0	1	1
SW	6	1	1	1	5	0	4	1	1	0	5	3	1	1	2	0
Total	26	21 (81%)	14 (54%)	4 (15%)	22 (85%)	2 (8%)	17 (65%)	11 (42%)	10 (38%)	0	19 (73%)	19 (73%)	4 (15%)	3 (12%)	10 (38%)	10 (38%)

**Houston, TX**																
MD	2	2	2	2	2	0	1	0	1	1	2	0	0	0	0	1
PA	1	1	1	0	1	0	0	1	1	0	1	0	0	0	0	0
Res	1	1	1	0	1	0	0	1	1	0	1	0	0	0	0	1
NP	3	3	3	0	3	0	0	1	1	1	2	2	0	0	1	0
RN	4	4	2	2	3	2	1	2	2	3	2	3	2	1	2	3
LVN	2	2	0	0	2	1	1	2	0	0	1	1	0	0	1	1
OT	1	1	1	0	1	0	0	1	0	1	0	0	0	1	0	1
PT	1	1	1	0	1	1	0	1	0	1	0	1	0	0	0	1
SW	3	2	1	0	1	0	1	1	1	0	1	1	1	1	0	1
Total	18	17 (94%)	12 (66%)	4 (22%)	15 (83%)	4 (22%)	4 (22%)	10 (56%)	7 (39%)	7 (39%)	10 (56%)	8 (44%)	3 (17%)	3 (17%)	4 (22%)	9 (50%)

**Total**	44	38 (86%)	26 (59%)	8 (18%)	37 (84%)	6 (14%)	21 (48%)	21 (48%)	17 (39%)	7 (16%)	29 (66%)	27 (61%)	7 (16%)	6 (14%)	14 (32%)	19 (43%)

* 'other' includes: patient depression, decreased function, lack of provider time, no place to exercise, wait time for care, no caregiver, patient or caregiver denial, problems with drug seeking behaviors

^ 'other' includes: patients need to be encouraged and empowered, anger management, work on self-esteem and confidence, need to distribute BP machines and pedometers, educate family members, allow for nursing follow up after discharge

MD, Medical Doctor

PA, Physicians Assistant

Res, Resident

NP, Nurse Practitioner

RN, Registered Nurse

LVN, Licensed Vocational Nurse

OT, Occupational Therapist

PT, Physical Therapist

RT, Recreation Therapist

SW, Social Worker

Identified needs included: improved patient and caregiver compliance; standardization of a stroke risk factor reminder, checklist, or approach; a way to refer to resources and services within the VA; better education to the providers regarding risk factors and warning signs; and improved administrative support. A summary of the emergent themes is available in Table 4.

**Table 4:** Summary of emergent themes from the needs assessment

Interview Topics	Supporting Themes	Indy N = 28	Houston N = 19
Current Provider Roles	Current roles of the provider to prevent a second stroke	81%	94%
	Working with or referring to other professionals or VA programs to prevent a second stroke	54%	66%
	Working with the patient, family, or caregiver to prevent a second stroke	15%	22%

Barriers and Supports to Secondary Stroke Risk Factor Management	Patient adherence/motivation/or set in their ways	85%	83%
	Provider lacks the knowledge or training to assist in secondary stroke risk factor management	8%	22%
	Level of support from the administration (barrier/support)	65%/15%	22%/41%
	Other: factors and characteristics such as poor adherence, decreased motivation, patients not wanting to change, and patients not taking responsibility for their self, depression, cognition, stroke severity, reading/education level, family relationships	42%	56%
	Patient lacks the cognition, education, knowledge, training, comfort to assist with prevention of a second stroke	38%	39%
	Patient transportation	0%	39%

Suggestions on how to Enhance Secondary Stroke Risk Factor Management Throughout the Continuum of Care	Desired resources: staff/provider education, handouts and pamphlets, standard training and discharge list, videos, support groups	93%	70%
	Training about what resources are available in the VA system, how to refer	38%	41%
	Timing of stroke risk factor management is important	30%	41%
	Other: important aspects of care: empowerment and encouragement of the patient, blood pressure machines, increased time with patient specifically for secondary stroke prevention information and training, and time to work with the family.	38%	65%

The majority of providers at both facilities (Indianapolis, 85% and Houston 82%) endorsed the fact that improved patient and caregiver compliance is important in managing health after stroke. Providers discussed less then optimal patient compliance and motivation to change as well as reasons for decreased compliance: depression; cognition; stroke severity; reading ability; transportation; and family relationships. An occupational therapist (OT) talked specifically about lack of compliance in following rehabilitation and diet recommendations once the patients are discharged into the home:

'... I feel like [diet] is a big component. It seems that if they...are not too compliant...what I've recommended does not make that big of an impact. In OT, we try to remind them how to incorporate their good diet, say when we do cooking and we turn to what they are going to be doing at home. We try to remind them and to incorporate their good diet into their selection, but they're still selecting the things that are bad for them despite what we've talked about.'

Multiple providers from different fields along the continuum of care suggested a need of a more standardized approach to secondary stroke prevention, including a systematic check-off list in the electronic medical records during the hospitalization. Specifically, a nurse was asked about provider training regarding stroke risk factors and stated:

'Standardization...it shouldn't be up to the physicians, like recognition, skills, knowledge ... because we get new doctors all the time...Everybody documents everything a little bit differently...but it should be like a math equation. It shouldn't be up to coincidence.'

Additionally, providers indicated that they worked with others in the VA facility or referred patients to other local community services or programs to assist in risk factor management (Indianapolis, 52%, and Houston, 68%). However, providers at both facilities discussed making patient referrals to highly visual VA services that cover common risk factors of smoking and diabetes; but many commented on needing to know about other available services and how to officially refer a patient to such services. For example, a resident was asked about the MOVE program (a VA nationally implemented exercise and nutrition program) and stated:

'No. I don't even know what that is. Why, why don't I know about this? It's frustrating to me that I don't know about this...But if I knew about them, I would be much more inclined and willing to use them. I just don't know about them. And I'm embarrassed that I don't, but I just don't have time to come into a place as a resident and say, 'Ok, I need to go do my homework, and find out exactly what my options right now.''

Thus, providers suggested a need to be educated on all locally available programming that addresses stroke risk factors. They need to know how they and patients can access it. Multiple providers also discussed needing some education regarding stroke risk factors and warning signs. Some providers talked about wanting to be more comfortable in talking about some risk factors, such as patient obesity. One doctor discussed discomfort with talking about obesity, but also provided a solution:

'...They don't like to talk about weight, [so] you avoid it. Then, they are not going to lose weight...I thought it was too sensitive to talk about weight...I found out that it took longer for them to lose the weight... So now I've found an indirect way to overcome it, by printing out weight graphs, and then use it to discuss with them. I give them BMI charts, so they are able to see for themselves. In fact, I've had patients tell me 'based on this weight, I'm obese.' Or 'based on this weight, I'm morbidly obese.' It becomes easier to then discuss. But when I used to avoid discussing this, it took a long time, and we failed quite a lot.'

Some providers discussed a need for additional administrative support to be able to implement a stroke prevention program. Many providers reported a lack of time to do as much as they would have liked to with patients to prevent a second stroke. Others felt that they needed resources, such as handouts and pamphlets, to best educate patients. However, others reported that stroke prevention had not been made enough of a priority in the hospital or a specific service and this barrier differed by site where providers in Indianapolis were more likely to endorse the idea that they did not receive the necessary support from administration (65% versus 35%).

We used the results of this needs assessment to plan the TOOLS program.

#### Step 1: Matrix of proximal program objectives

The planned intervention focused on adapting local tools to enable providers to systematically deliver secondary stroke prevention. We used the evidence-based guidelines of secondary stroke prevention to operationalize the components of secondary stroke prevention. Using these guidelines, we created proximal program objectives at the provider and organizational level and completed Step 1 of IM.

Step one of IM is to develop proximal program objectives, illustrated in a matrix of cells that include the intersection of behavioral or environmental proximal performance objectives (rows of table) with specified determinants (columns of table) (tables found in Additional File 1 Step 1) [2]. Determinants are personal and external factors that may influence outcomes. Each cell typically contains a statement, or a learning or change objective, regarding what needs to be learned related to this determinant to achieve the proximal performance objective.

Specifically, our proximal performance objectives were based upon the secondary stroke guidelines and included the following: assess patient stroke risk factors during hospitalization for stroke; order lab tests as needed; prescribe appropriate medications to manage risk factors; educate patients about stroke risk factor education; refer patient to local programs that address stroke risk factors; and motivate patient to modify lifestyle. These proximal performance objectives were crossed in the matrix with secondary stroke prevention delivery determinants. The determinants are based on the chronic care model and include: community resources for stroke risk management; patient self-management; health system organizational promotion of stroke risk factor management; delivery system design; decision support; and clinical information systems. Finally, change objective statements (*i.e*., the expected changes in the behavior and environment) were identified and added. The change objective statements were then used to guide us in the development of the TOOLS program. The proximal performance objectives, determinants, and subsequent change objective statements for the TOOLS program can be found in Additional File 1 Step 1.

#### Step 2: Selection of theory-based intervention methodologies

Bartholomew states that the goal of IM Step 2 is to use a conceptual model or theory to guide the identification of appropriate intervention methods and delivery strategies of these methods that are matched to the objectives stated in Step 1 [2]. A theoretical framework or model can be thought of as a supporting technique or process that influences change in the determinants identified in Step 1. We then used the components of the model to operationalize intervention components and implementation strategies.

For the TOOLS program, we reviewed the literature and chose the elements of the chronic care model [35] that fosters high-quality chronic disease care and applied them to secondary stroke prevention care. Given that secondary stroke care spanned inpatient and outpatient care services and targeted both the providers and patients, we believed the chronic care model elements were comprehensive. The elements are: clinical information systems support, delivery system design, decision support, self-management, and community resource access. For the implementation strategies, we incorporated the components of the theory of planned behavior [36] and specifically utilized strategies involving subjective norms/social persuasion for provider change strategies; and perceived behavior control/self-efficacy and goal setting facilitation for patient change strategies. In Additional File 1 Step 2, we identify both practical strategies to reach the objectives of Step 1 and suggestions that were derived from the provider semi-structured interviews completed with the needs assessment. An example of a provider suggestion that is supported by our conceptual model is that providers at both facilities suggested the development of a standardized checklist to ensure that each stroke survivor received the proper information and training to prevent a second stroke at discharge. This is supported through the model component of system design. See Additional File 1 Step 2 for additional examples.

#### Step 3: Design and organization of the TOOLS program

Step 3 of IM includes designing and organizing the program to be implemented. Following Bartholomew's recommendations, we used the results of the needs assessment, the generation of theoretical-based and practical strategies from the literature and the targeted users (IM Steps 1 and 2) to design and organize the TOOLS program in Step 3 (See Additional File 1 Step 3). We used the interviews to determine needs, as well as to discuss proposed strategies to assess the acceptability of the program, and to gain provider suggestions for implementation of the program. Main themes that emerged from the interviews included the need or desire for the following programs and strategies: standardized provider check-off list or discharge check-off list and clinical reminders; training and education regarding local resources and referral to such resources; provider stroke risk factor and prevention education; stroke support groups; peer programs; materials for patient education; and administration support. The resultant program included programming for both providers and veterans with stroke. See Table 2 and Additional File 1 Step 3 for a summarization of the recommendations and next action Steps that were derived from the interviews and IM. We specifically address some of the activities below.

Patient and caregivers factors, characteristics, and compliance impact prevention and lifestyle choices. Because prevention includes lifestyle change, some providers discussed the need to work with the patient, family members, and caregivers to best facilitate patient secondary stroke prevention. A doctor talked about the benefits of including family members into risk factor management:

'I found out that involving family helps a lot, because I found out some of the patients don't tell family. By family, I mean close family, the spouse, and the children. The children don't even know that the father is diabetic or has cholesterol problem. So when I involve them, some of the children, I find that they are more aware of the medical relationship between smoking and cholesterol.'

We implemented multiple activities to help provide a standardized approach to secondary stroke prevention. For example, we helped to develop a standard information packet that included handouts and pamphlets addressing the risk factor modification that is now given to all patients with stroke or TIA by a specified nurse prior to hospital discharge.

Interestingly, providers from both facilities (Indianapolis, 15%, and Houston, 24%) were interested in the development of a discharge template or check-off to ensure completion of secondary stroke prevention education and training. Due to this need, we developed a stroke risk factor checklist poster based on the guidelines that were placed in the neurology workstations at both sites and has been requested in an electronic format that is in progress.

An important concept arose when talking about available VA support and resources. Many providers were not aware of existing services and programs, and often did not know how to refer patients to risk factor management programs at their local facility, such as the MOVE (VA weight loss) program or stress management clinics. In order to address this important issue, and because people discussed the need for a more systematic approach to risk factor management at the facility level, we created a stroke risk factor 'prescription pad' (see Additional File 2). This prescription pad can be used by any VA provider to identify and 'prescribe' appropriate resources for each of the stroke risk factors and contact information at their local facility. For example, if someone is diagnosed with high BP, they can be sent to the VA hypertension clinic (phone number, day, and room information are provided), and/or they can receive home monitoring instructions and recommendations. If they are noted as having weight control issues (or obese), they are referred to the MOVE weight loss program (coordinator, phone number, and room number are provided). We have received positive feedback from the clinicians on this prescription pad and providers have subsequently requested the pad be transferred into an electronic order and that is a work in progress.

Because many providers discussed not necessarily having the knowledge or training to address the stroke risk factor modification, we provided standard training and education regarding patient motivational interviewing and goal setting to foster behavior change and support. We included role playing as part of this training (script available upon request). We also distributed materials and handouts for these providers to disseminate to patients and caregivers.

Because stroke support groups were mentioned by multiple providers at each facility, we have commenced with a monthly local stroke support group. Activities have included yoga, nutrition, stress management, finances after stroke, and caregiver support. Others talked about the importance of empowering the patient, teaching them to ask questions and encouraging them to make lifestyle changes and to be proactive. Multiple other providers talked about the need for BP machines. Previously, BP machines were easily issued to veterans who needed to control their hypertension, this is no longer the case and many providers would like to see this benefit returned. However, to fulfill this need through the TOOLS program, we are able to issue BP machines on site for teaching purposes and provided information to the patients for purchasing if interested. Additionally, we are able to provide pedometers, ergometers, resistance exercise bands, and/or a 10-minute relaxation CD for patient education and risk factor modification..

As self-management is an integral piece of the chronic care model [35] and discussed in our patient focus groups [37], we also planned program components with both the provider and the veterans to enhance self-management of stroke risk factors. We again trained the providers to use the prescription pad to refer veterans to community resources, but we also taught providers motivation interviewing and goal-setting techniques. This was to prepare the provider to begin discussions about stroke risk factor management. Additionally, we included training for the rehabilitation therapists to incorporate a stroke risk factor management goal for every patient with stroke or TIA. We also implemented self-management training for veterans to learn goal-setting techniques to modify his stroke risk factors to reduce his risk for secondary strokes.

Finally, we also specifically asked stroke survivors about existing programs for secondary stroke prevention. We asked care providers about the American Heart Association 'peer to peer' program, where a volunteer who has survived a stroke works with a patient with a new stroke. Both patients and their caregivers were excited about the support and guidance a fellow stroke survivor could provide. Stroke survivors repeatedly reported the desire to be around other stroke survivors who could relate to the functional limitations and role-functioning changes. The peer volunteer is a fellow stroke survivor and used as a support network to help guide the new stroke survivor through the process of stroke recovery. The majority of providers (65%) encouraged the use of this program and talked about how veterans often feel a connection to one another and that we should try to use this connection to enhance care. Thus we have included this in the TOOLS programming.

#### Step 4: Adoption and implementation of the TOOLS program

Prior to adoption and implementation of the TOOLS program, we locally tailored the intervention as per local needs and interests. For example, each site utilized a different self-management program with a local delivery schedule that fit into their healthcare system. We then fed back the program to a panel of local experts (*i.e*., chiefs of neurology), leaders from different clinical services, and some levels of administration at each facility to gain feedback prior to implementation. We also secured a 'clinical champion' at each facility to help assist with the implementation of the TOOLS program, and importantly to help sustain it after the end of the study funding.

Step 4 of IM includes the adoption and implementation plan for the program in the prescribed setting and is vital to ensure delivery of the program [2]. Step 4 includes complex tracking of each aspect of the program and working with providers and administration to address any issues prior to roll out of the program. For TOOLS, this includes complex tracking of how each of the intervention components are delivered and used by the veteran or the provider, where they are used, and the delivery format (via group, individual, face, telephone, or electronic). We also include our patient self-management checklist where we are able to document which self-management activities the patient engaged in to manage their stroke risk factors. (Additional File 1 Step 4).

#### Step 5: Monitoring and program evaluation

Monitoring and evaluation of the program is the last Step of IM. This evaluation uses the planned products of other IM Steps to evaluate the process and the effect of the program [2]. It is necessary to plan for the evaluation of the program, and it should include reflection on the determinants, provider and patient behaviors, and health outcomes. Bartholomew and colleagues indicate that IM allows for thoughtful formative evaluation to best evaluate both process and effect of the program and whether changes need to be made [2].

Our program monitoring and evaluation can be found in Additional File 1 Step 5. It includes primary and secondary outcomes, evaluation of change both at the provider and patient level, utilized measures, the time it takes to complete the individual assessments, and a schedule of assessments at baseline, three months, and six months post-intervention. At the provider level, we were interested in determining whether there was lifestyle or medication management counseling, or specific stroke prevention goals in the rehabilitation notes. This will all be completed through medical record reviews. At the patient level, we will assess stroke quality of life, stroke severity, physical functioning, depression, self-efficacy, knowledge of stroke signs and risk factors, and outcome expectations through self-report and medical record review.

## Discussion

Similar to previous health promotion programs, we used IM to guide the development and implementation plan of an evidence-based intervention targeting secondary stroke prevention. IM provides a planning template for incorporating theoretical components, practical strategies, evidence-based components from the literature, and direct input from the targeted user groups. By conducting a needs assessment at both sites, we found that most VA health providers are interested in engaging in secondary stroke prevention; however, they needed better resources, training, and implementation guidance. Moreover, their needs were different at each facility and IM allowed us to tailor the intervention to each.

While this paper is not reporting the performance rate on secondary stroke indicators of care, we did query clinical providers on their current practices according to the VA/DoD and the American Stroke Association guidelines related to secondary stroke risk factor management and prevention to identify best practices and gaps. While the majority of our interviewed providers indicated that they participated in secondary stroke prevention at some level, many talked about referring to other healthcare providers or not being competent to provide such information. This parallels a recent study we completed where we surveyed all occupational and physical therapists in the Midwest region. Therapists often indicated that they were likely to refer patients to other healthcare providers, or that secondary stroke prevention was not part of their role as a therapist [40]. We also found that therapists were not aware of VA stroke rehabilitation guidelines, indicating that part of the TOOLS intervention will need to be basic education regarding guideline compliance and education about stroke prevention, risk factors, and stroke warning signs. From our interviews, the rehabilitation therapists specifically discussed interest in learning about how to include secondary stroke prevention in goal writing. This is important because goal writing has been called the 'essence of rehabilitation,' and we believe it may be used as a modality to change rehabilitation practice as it is related to risk factor management [41].

Our study also identified provider needs to better support secondary stroke prevention. Multiple providers discussed patient adherence with medication, physical activity, and lifestyle change. Rimmer *et al*. assessed the barriers to physical activity for people with stroke and found that the four most common barriers included: cost of programming, not knowing about a local fitness center or where to exercise, lack of transportation, and not knowing how to exercise [42]. Therefore, to enhance adherence in the TOOLS study, it is essential for us to tailor the intervention to each individual patient to best accommodate their needs and enhance secondary stroke prevention outcomes. Thus, we are encompassing self-management strategies to modify stroke risk factors [43].

Once we have completed the TOOLS program at both sites, we will complete the evaluations of Step 5 and focus groups of veterans and providers. We will use these focus groups to better understand how the TOOLs program altered care and self-management of stroke risk factors. We will also seek information on how to better adapt it for both veterans and providers for future implementation.

## Summary

We completed IM to develop an evidence-based program to systematically deliver at two different facilities. The use of IM has allowed us to determine our goals, the determinants, change objectives, practical strategies, evaluation of the program, and the program itself. This will guide us as we implement the program into the two pre-determined facilities but also as we move forward into different settings.

## Competing interests

The authors declare that they have no competing interests.

## Authors' contributions

All authors were involved with drafting and reviewing the manuscript. Specifically, AS drafted the manuscript as the primary author, completed revisions with TD, helped complete study participant interviews, and participated in the design of the study and the development of the interviews. JA participated in the conception and design of the study, data collection, and made substantial contributions to the manuscript. TK participated in the conception and design of the study and is the attending neurologist for the study at Houston site. LW is the attending neurologist for the study at the Indianapolis site and participated in the conception and design of the study. TD is the PI of the study, participated in the conception and design of the study, helped with data collection and development of interviews, and made substantial contributions to the manuscript and revisions and developed the matrix. All authors read and approved the final draft.

## Supplementary Material

Additional file 1 **TOOLS Secondary Stroke Prevention, Intervention Mapping, Steps 1-5**. The additional file includes specific information for each of the Intervention Mapping Steps. All steps are included in table format. Specifically we include: Intervention Mapping, Step 1: Secondary stroke prevention program matrix of proximal program objectives at the provider and organizational level. Intervention Mapping, Step 2: Theoretical and practical strategies to systematically deliver secondary stroke prevention matched to proximal program objectives. Intervention Mapping, Step 3: Program design to tailor a stroke secondary prevention program - implementation intervention Intervention Mapping, Step 4, Adoption and implementation plans. Intervention Mapping, Step 5, Evaluation of intervention impact. Click here for file

Additional file 2 **Prescription Pad**. The additional file includes an example of the 'prescription pad' we used to help management of stroke risk factors for our specific VA hospital. Click here for file
