# Supplementary material for: Using intervention mapping to develop and adapt a secondary stroke prevention program in Veterans Health Administration medical centers
Source: Implement Sci. 2010 Dec 15;5:97. doi: 10.1186/1748-5908-5-97 (PMC3057184; doi:10.1186/1748-5908-5-97)
Supplement: Additional file 1 — TOOLS Secondary Stroke Prevention, Intervention Mapping, Steps 1-5. The additional file includes specific information for each of the Intervention Mapping Steps. All steps are included in table format. Specifically we include: Intervention Mapping, Step 1: Secondary stroke prevention program matrix of proximal program objectives at the provider and organizational level. Intervention Mapping, Step 2: Theoretical and practical strategies to systematically deliver secondary stroke prevention matched to proximal program objectives. Intervention Mapping, Step 3: Program design to tailor a stroke secondary prevention program - implementation intervention Intervention Mapping, Step 4, Adoption and implementation plans. Intervention Mapping, Step 5, Evaluation of intervention impact. [file 1748-5908-5-97-S1.DOC]

**Additional file**

**TOOLS Secondary Stroke Prevention (Damush *et al.* from HSRD IIR Adapting Tools for Secondary Stroke Prevention In VA)**

**Intervention Mapping, Step 1: Secondary stroke prevention program matrix of proximal program objectives at the provider and organizational level**

| **Secondary stroke prevention delivery determinants based upon chronic care model** | | | | | | |
| --- | --- | --- | --- | --- | --- | --- |
| **Provider Performance Objectives** | **Community Resources for Stroke Risk Management** | **Patient Self-Management** | **Health System Organizational Promotion of Stroke Risk Factor Management** | **Delivery System Design** | **Decision Support** | **Clinical Information Systems** |
| **Assesses patient stroke risk factors during hospitalization for stroke** | Access to local resources available to assess stroke risk factors | Communicates the patient’s risk factors for stroke to patient | Neurology service at facility promotes stroke risk factor assessment | Work flow of discharge planning includes the systematic delivery of stroke risk factor assessment | Practices within the guidelines by the Joint Commission, VA/DoD and American Stroke Association to assess stroke risk factors | Records the results of assessment in a template in the patient medical records |
| **Orders lab tests as needed** | Access to patient materials on laboratory tests and interpretation of results | Communicates with patient on need for tests and on patient results | Neurology service at facility orders lab tests per guideline care and prescribes treatment to modify risk factors based on results | Orders lab tests per guideline care; System alerts provider of lab results; Provider prescribes therapy based on results | Practices within the guidelines by the Joint Commission, VA/DoD and American Stroke Association to order labs | Documents the order for labs and results of lab tests |
| **Prescribes appropriate medications to manage risk factors** | Accesses and provides patient education materials on medications prescribed for stroke risk factor management | Educates the patient on the benefits of the medication prescribed, prescribed dosage, medication adherence, and potential side effects | Healthcare system provides medication prescribed to veterans with stroke | Medication reconciliation prior to hospital discharge is standard practice with pharmacist for veterans hospitalized with stroke | Accesses clinical algorithms/pocket cards based on guideline care for patients with stroke | Utilizes clinical reminders and electronic templates to prescribe medications for stroke patients as appropriate; |
| **Educates patient about stroke risk factor reduction** | Accesses and provides patient with appropriate and relevant stroke risk factor education materials | Discusses goal setting to modify stroke risk factors; provides feedback on patient efforts to modify risk factors | Dedicated staff members are assigned the responsibility of patient education on stroke risk factors | Neurologists and nurses document education provided on the specific risk factors present in the patient prior to discharge | Clinical reminders and support materials are available for staff | Patient education materials are available on demand at time of patient exam |
| **Refers patient to local programs that address stroke risk factors** | Recommends MOVE program; smoking cessation classes and support; nutritional counseling; diet classes; anger management classes; physical activity in the community; diabetes management; | Refers patient to diabetes self management courses; refer to local and community support groups; | Health care system provides support programs to reduce risk factors; Providers are aware of how to access programs for their patients | Access to local programs is readily available for patients; | Patient information is available on Myhealthevet | Social workers; Rehab therapists; Doctors; nurses have access to locally available support programs for stroke patients |
| **Motivates patient to modify lifestyle** | Writes orders for home equipment to support patient lifestyle changes (*e.g.*, pedometers, blood pressure machines); accesses information on local programs | Applies motivational interviewing to motivate patient to modify lifestyle; negotiates lifestyle change efforts with patient | The organization culture supports the providers time to spend on patient lifestyle modification | Patient lifestyle modification is part of the stroke discharge process | Utilizes pocket cards/graphical displays to motivate patient lifestyle modification | The medical record includes electronic templates to document lifestyle modification efforts and progress |

Intervention Mapping, Step 2: Theoretical and practical strategies to systematically deliver secondary stroke prevention matched to proximal program objectives

|  | **Theoretical Strategies** | **Practical Strategies** | **Provider Suggestions from Semi-Structured Interviews** |
| --- | --- | --- | --- |
| **Provider Performance Objectives** | Theory of Planned Behavior |  |  |
| **Assesses patient stroke risk factors during hospitalization for stroke** | Perceived Social Norms - clinical champion promotes; added into annual competency evaluation  Attitudes, Beliefs, Values - training  Self-efficacy/control - role playing to improve skills; vicarious/peer modeling  Behavioral intentions - assess intentions/commitment to perform | In the discharge planning template, a stroke risk factor assessment template is included; A computer reminder for stroke risk factor assessment is alerted at time of stroke discharge planning | Suggest systematic provision of a check off list in the medical records during hospitalization |
| **Orders lab tests as needed** | Perceived Social Norms - clinical champion promotes; added into annual competency evaluation  Attitudes, Beliefs, Values - training  Self-efficacy/control - role playing to improve skills; vicarious/peer modeling  Behavioral intentions – assess intentions/commitment to perform | Provider receives a clinical reminder to order specific lab tests upon patient admission for stroke; Provider systematically accesses clinical templates to order lab tests; Provider receives clinical alert of lab tests results | Providers already use in electronic medical records |
| **Prescribes appropriate medications to manage risk factors** | Perceived Social Norms - clinical champion promotes; added into annual competency evaluation  Attitudes, Beliefs, Values - training  Self-efficacy/control- role playing to improve skills; vicarious/peer modeling  Behavioral intentions – assess intentions/commitment to perform | Provider systematically accesses clinical templates to prescribe appropriate medications per clinical guideline care and results of lab tests; Accesses decision support on appropriate prescriptions available and potential interactions | Providers already use in electronic medical records |
| **Educates patient about stroke risk factor reduction** | Self-efficacy to motivate patients to change their lifestyles – training, role playing to improve skills; vicarious/peer modeling through follow up sessions | Provider provides on demand patient education materials about specific risk factor relevant to the patient; Provider answers patient questions; Pharmacists provides medicine reconciliation prior to hospital discharge to review all prescribed medications and educate patient about side effects and answer patient questions | Provide clinicians with educational materials and handouts for both the patient and the caregiver |
| **Refers patient to local programs that address stroke risk factors** | Perceived social norms – clinical champions promotes referrals  Self-efficacy – improve the MD confidence to make appropriate referrals in VA and the community. | Provider discusses local program options available for the patient to modify risk factors and communicates his/her recommendation for the patient to participate | Provide clinical staff with updated information on all the local available programs addressing the stroke risk factors and how their patients may access it. Would like a stroke support group to send their patients with stroke for such support |
| **Motivates patient to modify lifestyle** | Self-efficacy to motivate patients to change their lifestyles – training, role playing to improve skills; vicarious/peer modeling through follow up sessions | Provider is trained in motivational interviewing and goal setting; Provider applies motivational interviewing principles to assist patient plan lifestyle modifications; Provider collaborates with patient to set goals to change health behaviors; | Providers need training on starting the conversation with their patients on lifestyle modification and tools to support their patient efforts. Needs support for motivating patients to change. Recognizes the importance of family support on their patients’ health behaviors |

Intervention Mapping, Step 3: Program design to tailor a stroke secondary prevention program – clinical intervention

| **Provider**  **Performance**  **Objectives** | **Community**  **Resources for Stroke Risk Management** | **Patient Self-Management** | **Health System Organizational Promotion of Stroke Risk Factor Management** | **Delivery System Design** | **Decision Support** | **Clinical Information Systems** |
| --- | --- | --- | --- | --- | --- | --- |
| **Assesses patient stroke risk factors during hospitalization for stroke** | Provider accesses local resources available to assess patient stroke risk factors | Communicates patient’s specific stroke risk factors and begins the conversation on how to modify and conveys its importance | Neurology service endorses stroke risk factor assessment during hospitalization for stroke as usual practice | Nurse systematically assesses patient stroke risk factors during hospitalization and documents in electronic medical records | Guideline care for stroke risk factor management provided on posters and placed at neurology workstations | Electronic medical record templates created |
| **Orders lab tests as needed** | Access to patient education materials on lab tests results | Provider communicates lab test results with patient | Neurology service endorses lab tests orders during hospitalization for stroke as usual practice | Neurology electronic medical record templates systematically include orders for lab tests for stroke patients | Guideline care for stroke risk factor management provided on posters and placed at neurology workstations | Utilizes existing electronic medical record templates |
| **Prescribes appropriate medications to manage risk factors** | Access to patient education materials on medications | Includes existing practice of Pharmacist providing medicine reconciliation prior to discharge to review all medicines prescribed, their instructions, side effects and answer patient questions | Neurology service endorses the prescription of medications designed to reduce stroke risk upon discharge for stroke as usual practice | Neurology electronic medical record templates systematically include orders for prescriptions for stroke risk factor management | Guideline care for stroke risk factor management provided on posters and placed at neurology workstations | Utilizes existing electronic medical record templates |
| **Educates patient about stroke risk factor reduction** | Materials/Handouts provided for providers to deliver to patients on specific risk factors | American Stroke Association Peer Visitor Program implemented to support veteran with stroke; | Nurses are assigned the responsibility of patient education on stroke risk factor management | The documentation of nurse risk factor education is required prior to hospital discharge | On demand education is available for staff | Modified existing patient education nurse template to document specific risk factor education delivered documentation |
| **Refers patient to local programs that address stroke risk factors** | Prescription pad created for local programs for each stroke risk factor with instructions on how patients may access | Stroke Self Management program provided for patients in hospital/after discharge; | Healthcare organization provides support programs to reduce stroke risk factors (*e.g.*, smoking cessation; diet modification) | Prescription pad made available at neurology resident workstation | Prescription pad outlines appropriate programs by stroke risk factor | Created electronic version of risk factor prescription pad |
| **Motivates patient to modify lifestyle** | Provided Provider training on community resources available | Provided Provider training on motivational interviewing and goal setting | Healthcare organization provides support devices for lifestyle modification (*e.g.*, pedometers from prosthetics) | Incorporates lifestyle modification into patient clinical goals | Accesses information on MyhealthEvet | Created patient goal setting checklist for stroke self management |

**Intervention Mapping, Step 3: Program design to tailor a stroke secondary prevention program – implementation intervention**

|  | **Theoretical Strategies** | **Implementation Strategies** |
| --- | --- | --- |
| **Provider Performance Objectives** | Theory of Planned Behavior | Operationalized |
| **Assesses patient stroke risk factors during hospitalization for stroke** | Perceived Social Norms - clinical champion promotes stroke risk factor assessment; added into annual competency evaluation  Attitudes, Beliefs, Values - training  Self-efficacy/control- role playing to improve skills; vicarious/peer modeling  Behavioral intentions – assess intentions/commitment to perform | Chief of Neurology assigns neurology nurse to assess stroke risk factors of all patients admitted with stroke, document results in a electronic discharge planning template, and provide a education packet that addresses the stroke risk factor prior to discharge. Research team provides training, role playing and support materials to clinicians. Assesses attitudes and intentions to perform in next 90 days. Post performance objectives and guideline care at neurology workstations where discharge planning occurs. |
| **Orders lab tests as needed** | Perceived Social Norms - clinical champion promotes; added into annual competency evaluation, provides feedback on performance  Attitudes, Beliefs, Values - training  Self-efficacy/control- role playing to improve skills; vicarious/peer modeling  Behavioral intentions – assess intentions/commitment to perform | Measures performance and provides regular feedback on quality of performance to providers. Provides training to clinicians and assesses behavioral intentions. |
| **Prescribes appropriate medications to manage risk factors** | Perceived Social Norms - clinical champion promotes; added into annual competency evaluation  Attitudes, Beliefs, Values - training  Self-efficacy/control- role playing to improve skills; vicarious/peer modeling  Behavioral intentions – assess intentions/commitment to perform | Provider systematically accesses clinical templates to prescribe appropriate medications per clinical guideline care and results of lab tests; Accesses decision support on appropriate prescriptions available and potential interactions. Measures performance and provides regular feedback on quality of performance. Provides training, and evaluates intentions to perform over next 90 days |
| **Educates patient about stroke risk factor reduction** | Self-efficacy to motivate patients to change their lifestyles – training, role playing to improve skills; vicarious/peer modeling through follow up sessions | Provides on demand patient education materials about specific risk factor relevant to the patient; Provides training on talking to patients about stroke risk factors and the social influence of the provider. Provides training and role playing on addressing pt objections and barriers. Pharmacists (practical strategy) provide medicine reconciliation prior to hospital discharge to review all prescribed medications and educate patients about side effects and answer patient questions. |
| **Refers patient to local programs that address stroke risk factors** | Perceived social norms – clinical champions promotes referrals  Self-efficacy – improve the MD confidence to make appropriate referrals in VA and the community. | Provider is familiar with an update on local programs that address pt risk factors; discusses local program options available for the patient to modify risk factors and communicates his/her recommendation for the patient to participate; provides risk factor referral Rx pad to use with patients. |
| **Motivates patient to modify lifestyle** | Self-efficacy to motivate patients to change their lifestyles – training, role playing to improve skills; vicarious/peer modeling through follow up sessions | Provider is trained in motivational interviewing and goal setting –education; role playing/social modeling; Provider applies motivational interviewing principles to assist patient plan lifestyle modifications; Provider collaborates with patient to set goals to change health behaviors; |

Intervention Mapping, Step 4, Adoption and implementation plans

| **Chronic Care Model Factor** | **Intervention Component** | **Fidelity** | **Date(s)** | **Provider/**  **Instructor** | **Place**  **Houston/ Indianapolis**  **Clinic/Dept/ Floor** | **Contents** | **Meeting**  **Format:**  Group ❑  Individual ❑  Other ❑ | **Delivery Mode:**  Face ❑  Telephone ❑  Electronic ❑ |
| --- | --- | --- | --- | --- | --- | --- | --- | --- |
| 1. Community Resources for stroke risk management | Provider gave participant educational materials from community resources (*e.g.*, AHA/ASA) | Notes in electronic medical records  Yes ❑      No ❑ |  |  | Houston ❑  Indianapolis ❑  Clinic ❑  Department ❑  Floor ❑ |  | Group ❑  Individual ❑  Other ❑ | Face ❑  Telephone ❑  Electronic ❑ |
|  | Participant obtained community resources (*e.g.*, AHA/ASA materials) | Self-report  Yes ❑      No ❑ |  |  | Houston ❑  Indianapolis ❑  Clinic ❑  Department ❑  Floor ❑ |  | Group ❑  Individual ❑  Other ❑ | Face ❑  Telephone ❑  Electronic ❑ |
|  | Participant utilized community exercise programs (YMCA, other) | Sef-report  Yes  No |  |  | Houston ❑  Indianapolis ❑  Clinic ❑  Department ❑  Floor ❑ |  | Group ❑  Individual ❑  Other ❑ | Face ❑  Telephone ❑  Electronic ❑ |
|  | Participant enrolled in VA support program (*e.g.*, MOVE, smoking cessation, others) | Program logs;  Medical records |  |  | Houston ❑  Indianapolis ❑  Clinic ❑  Department ❑  Floor ❑ |  | Group ❑  Individual ❑  Other ❑ | Face ❑  Telephone ❑  Electronic ❑ |
| 2. Health System promotion of stroke risk management | JCAHO Guidelines  promoted | Yes ❑      No ❑ |  |  | Houston ❑  Indianapolis ❑  Clinic ❑  Department ❑  Floor ❑ |  | ❑ Inservice  ❑ Team mtg  ❑ Didactic  training  ❑ Other ___________ | Face ❑  Telephone ❑  Electronic ❑ |
|  | VA/DoD Guidelines  promoted | Yes ❑      No ❑ |  |  | Houston ❑  Indianapolis ❑  Clinic ❑  Department ❑  Floor ❑ |  | ❑ Inservice  ❑ Team mtg  ❑ Didactic  training  ❑ Other ___________ | Face ❑  Telephone ❑  Electronic ❑ |
|  | AHA/ASA guidelines  promoted | Yes ❑      No ❑ |  |  | Houston ❑  Indianapolis ❑  Clinic ❑  Department ❑  Floor ❑ |  | ❑ Inservice  ❑ Team mtg  ❑ Didactic  training  ❑ Other ___________ | Face ❑  Telephone ❑  Electronic ❑ |
| 3. Patient Self-Management for stroke risk reduction | ASA Peer Stroke Support  Other:_______ | Research database  Treatment Delivery  Participant invited to attend  Yes ❑      No ❑ |  |  | Houston ❑  Indianapolis ❑  Clinic ❑  Department ❑  Floor ❑ |  |  | Face ❑  Telephone ❑  Electronic ❑ |
|  | 1^st^ meeting | Yes ❑      No ❑ |  |  |  |  |  | Face ❑  Telephone ❑  Electronic ❑ |
|  | 2^nd^ meeting | Yes ❑      No ❑ |  |  |  |  |  | Face ❑  Telephone ❑  Electronic ❑ |
|  | 3^rd^ meeting | Yes ❑      No ❑ |  |  |  |  |  | Face ❑  Telephone ❑  Electronic ❑ |
|  | 4^th^ meeting | Yes ❑      No ❑ |  |  |  |  |  | Face ❑  Telephone ❑  Electronic ❑ |
|  | 5^th^ meeting | Yes ❑      No ❑ |  |  |  |  |  | Face ❑  Telephone ❑  Electronic ❑ |
|  | 6^th^ meeting | Yes ❑      No ❑ |  |  |  |  |  | Face ❑  Telephone ❑  Electronic ❑ |
|  | Additional meetings | Yes ❑      No ❑ |  |  |  |  |  | Face ❑  Telephone ❑  Electronic ❑ |
| Provider counseling | Counsel on smoking cessation | Yes ❑      No ❑ |  |  | Houston ❑  Indianapolis ❑  Clinic ❑  Department ❑  Floor ❑ |  |  | Face ❑  Telephone ❑  Electronic ❑ |
|  | Counsel on AHA diet | Yes ❑      No ❑ |  |  | Houston ❑  Indianapolis ❑  Clinic ❑  Department ❑  Floor ❑ |  |  | Face ❑  Telephone ❑  Electronic ❑ |
|  | Counsel on exercise | Yes ❑      No ❑ |  |  | Houston ❑  Indianapolis ❑  Clinic ❑  Department ❑  Floor ❑ |  |  | Face ❑  Telephone ❑  Electronic ❑ |
|  | Discuss medications  -Statins  -ACE inhibitor  -Antithrombotics  -Antiplatets | Yes ❑      No ❑ |  |  | Houston ❑  Indianapolis ❑  Clinic ❑  Department ❑  Floor ❑ |  |  | Face ❑  Telephone ❑  Electronic ❑ |
| Case-management | Referral to existing nurse/social worker program | CPRS – medical records |  |  | Houston ❑  Indianapolis ❑  Clinic ❑  Department ❑  Floor ❑ |  |  | Face ❑  Telephone ❑  Electronic ❑ |
|  | Telehealth device monitoring | CPRS – medical records |  |  | Houston ❑  Indianapolis ❑  Clinic ❑  Department ❑  Floor ❑ |  |  | Face ❑  Telephone ❑  Electronic ❑ |
| 4. Delivery System | Local champion exists for stroke risk reduction | Yes ❑      No ❑ |  |  | Houston ❑  Indianapolis ❑  Clinic ❑  Department ❑  Floor ❑ |  |  |  |
|  | Referrals made by providers to VA resources | Yes ❑      No ❑ |  |  |  | ❑ Rehab  ❑ MOVE  ❑ PERC  ❑ Nutrition  ❑ Smoking Cessation  ❑ Other  ________ |  |  |
|  | Staff training for patient lifestyle counseling | Yes ❑      No ❑ |  |  | Houston ❑  Indianapolis ❑  Clinic ❑  Department ❑  Floor ❑ |  | ❑ Inservice  ❑ Team mtg  ❑ Didactic  training  ❑ Other ___________ |  |
|  | Nurse standing order for medication management for stroke risk reduction | Yes ❑      No ❑ |  |  | Houston ❑  Indianapolis ❑  Clinic ❑  Department ❑  Floor ❑ |  |  | Face ❑  Telephone ❑  Electronic ❑ |
|  | Dedicated clinic forms to flag stroke patients | Yes ❑      No ❑ |  |  |  |  |  |  |
| 5. Decision Support | Pocket cards provided | Yes ❑      No ❑ |  |  | Houston ❑  Indianapolis ❑  Clinic ❑  Department ❑  Floor ❑ |  |  |  |
|  | Preprinted/ electronic admission order sheets with checked boxes | Yes ❑      No ❑ |  |  | Houston ❑  Indianapolis ❑  Clinic ❑  Department ❑  Floor ❑ |  |  |  |
|  | Checked fasting lipids | Yes ❑      No ❑ |  |  | Houston ❑  Indianapolis ❑  Clinic ❑  Department ❑  Floor ❑ |  |  |  |
|  | Checked fasting glucose | Yes ❑      No ❑ |  |  | Houston ❑  Indianapolis ❑  Clinic ❑  Department ❑  Floor ❑ |  |  |  |
|  | Checked hemoglobin A1c values | Yes ❑      No ❑ |  |  | Houston ❑  Indianapolis ❑  Clinic ❑  Department ❑  Floor ❑ |  |  |  |
|  | Checked hypertension status | Yes ❑      No ❑ |  |  | Houston ❑  Indianapolis ❑  Clinic ❑  Department ❑  Floor ❑ |  |  |  |
|  | Calculated BMI | Yes ❑      No ❑ |  |  | Houston ❑  Indianapolis ❑  Clinic ❑  Department ❑  Floor ❑ |  |  |  |
|  | Medication Algorithms Provided | Yes ❑      No ❑ |  |  | Houston ❑  Indianapolis ❑  Clinic ❑  Department ❑  Floor ❑ |  |  |  |
|  | Prescribed hypertension meds as appropriate | Yes ❑      No ❑ |  |  | Houston ❑  Indianapolis ❑  Clinic ❑  Department ❑  Floor ❑ |  |  |  |
|  | Prescribed  Antithrombotic meds as appropriate | Yes ❑      No ❑ |  |  | Houston ❑  Indianapolis ❑  Clinic ❑  Department ❑  Floor ❑ |  |  |  |
|  | Prescribed Statins as needed | Yes ❑      No ❑ |  |  | Houston ❑  Indianapolis ❑  Clinic ❑  Department ❑  Floor ❑ |  |  |  |
|  | Educated patient on Stroke information –signs, symptoms, call 911 | Yes ❑      No ❑ |  |  | Houston ❑  Indianapolis ❑  Clinic ❑  Department ❑  Floor ❑ |  |  |  |
| 6. Clinical Information System | Electronic reminder for stroke guideline care in CPRS | Yes ❑      No ❑ |  |  | Houston ❑  Indianapolis ❑ |  |  |  |
|  | Developed local automated identifiers for stroke patients | Yes ❑      No ❑ |  |  | Houston ❑  Indianapolis ❑ |  |  |  |
|  | Developed automated clinical form to monitor performance | Yes ❑      No ❑ |  |  | Houston ❑  Indianapolis ❑ |  |  |  |

(Step 4 Continued) Patient Self-Management Checklist

| **Stroke Self-Management Course Overview** | **Week 1** | | | | | | | **Week 2** | | | | | | |
| --- | --- | --- | --- | --- | --- | --- | --- | --- | --- | --- | --- | --- | --- | --- |
|  | M | T | W | R | F | Sa | Su | M | T | W | R | F | Sa | Su |
| **DATE OF SESSION________** |  |  |  |  |  |  |  |  |  |  |  |  |  |  |
| Talked to my doctor about receiving physical or speech or occupational therapy |  |  |  |  |  |  |  |  |  |  |  |  |  |  |
| Attended my rehabilitation therapy appointments |  |  |  |  |  |  |  |  |  |  |  |  |  |  |
| Learned the warning signs of stroke |  |  |  |  |  |  |  |  |  |  |  |  |  |  |
| Discussed my fears about recovery |  |  |  |  |  |  |  |  |  |  |  |  |  |  |
| Talked to my doctor about depression |  |  |  |  |  |  |  |  |  |  |  |  |  |  |
| Practiced Rehab Exercises from therapist |  |  |  |  |  |  |  |  |  |  |  |  |  |  |
| Practiced Personal Exercise Program in handout |  |  |  |  |  |  |  |  |  |  |  |  |  |  |
| Practiced problem-solving |  |  |  |  |  |  |  |  |  |  |  |  |  |  |
| Used a pill box to keep track of medications |  |  |  |  |  |  |  |  |  |  |  |  |  |  |
| Took pills as my doctor recommended |  |  |  |  |  |  |  |  |  |  |  |  |  |  |
| Contacted a community resource:____________ |  |  |  |  |  |  |  |  |  |  |  |  |  |  |
| Became Active around the Home:____________ |  |  |  |  |  |  |  |  |  |  |  |  |  |  |
| Practiced Exercises from the Exercise and Daily Activity Book |  |  |  |  |  |  |  |  |  |  |  |  |  |  |
| Walked the dog |  |  |  |  |  |  |  |  |  |  |  |  |  |  |
| Walked in the community |  |  |  |  |  |  |  |  |  |  |  |  |  |  |
| Practiced Other Physical Activity:_____________ |  |  |  |  |  |  |  |  |  |  |  |  |  |  |
| Practiced Deep Breathing |  |  |  |  |  |  |  |  |  |  |  |  |  |  |
| Listened to Relaxation CD |  |  |  |  |  |  |  |  |  |  |  |  |  |  |
| Practiced Progressive Muscle Relaxation |  |  |  |  |  |  |  |  |  |  |  |  |  |  |
| Practiced Changing Negative thoughts to Positive |  |  |  |  |  |  |  |  |  |  |  |  |  |  |
| Ate healthy foods |  |  |  |  |  |  |  |  |  |  |  |  |  |  |
| Eliminated unhealthy foods from diet |  |  |  |  |  |  |  |  |  |  |  |  |  |  |
| Attended Smoking Cessation classes |  |  |  |  |  |  |  |  |  |  |  |  |  |  |
| Used smoking cessation aides |  |  |  |  |  |  |  |  |  |  |  |  |  |  |
| Stopped smoking |  |  |  |  |  |  |  |  |  |  |  |  |  |  |
| Reduced alcohol consumption |  |  |  |  |  |  |  |  |  |  |  |  |  |  |
| Stopped consuming alcohol |  |  |  |  |  |  |  |  |  |  |  |  |  |  |
| Tested my sugar level for diabetes |  |  |  |  |  |  |  |  |  |  |  |  |  |  |
| Used a salt substitute in my meals |  |  |  |  |  |  |  |  |  |  |  |  |  |  |
| Reduced salt in my meals |  |  |  |  |  |  |  |  |  |  |  |  |  |  |
| Stopped using salt in my meals |  |  |  |  |  |  |  |  |  |  |  |  |  |  |
| Brought my doctor a list of my health concerns |  |  |  |  |  |  |  |  |  |  |  |  |  |  |
| Asked my doctor questions |  |  |  |  |  |  |  |  |  |  |  |  |  |  |
| Asked my doctor for explanations |  |  |  |  |  |  |  |  |  |  |  |  |  |  |
| Measured my blood pressure |  |  |  |  |  |  |  |  |  |  |  |  |  |  |
| Weighed myself |  |  |  |  |  |  |  |  |  |  |  |  |  |  |
| OTHER: |  |  |  |  |  |  |  |  |  |  |  |  |  |  |
| OTHER: |  |  |  |  |  |  |  |  |  |  |  |  |  |  |
| OTHER: |  |  |  |  |  |  |  |  |  |  |  |  |  |  |
| OTHER: |  |  |  |  |  |  |  |  |  |  |  |  |  |  |

(Step 4 Continued) Stroke Peer Visitor Monitoring

**TRACKING YOUR PEER VISITS**

**Name of Patient, date of visit, place of visit, length of time of visit**

| **Patient Name** | **Visit Date** | **Place of Visit** | **Length of time of visit** | **Any special requests/needs** |
| --- | --- | --- | --- | --- |
|  |  |  |  |  |
|  |  |  |  |  |
|  |  |  |  |  |
|  |  |  |  |  |
|  |  |  |  |  |
|  |  |  |  |  |
|  |  |  |  |  |

Intervention Mapping, Step 5, Evaluation of intervention impact

| Outcome assessment protocol: Measures and schedule of administration | | | | | | | |
| --- | --- | --- | --- | --- | --- | --- | --- |
| **Domain** | **Source** | **Measure** | **Items** | **Time (min)** | **Schedule**  **BL 3 MO 6 MO** | | |
| **PRIMARY OUTCOMES** | | | | | | | |
| Stroke Risk Factor Management Provided by Staff/Providers | Medical Records | Medication Management  Lifestyle counseling/referral  [treat yes/no: dose, duration] | CPRS  Abstract |  | X | X | X |
| Stroke Risk Factor Management Behaviors | Survey | Lifestyle Behaviors  -Physical Activity  - Diet  -Smoking  -Alcohol  -Medication Adherence | 20 | 15 | X | X | X |
| **SECONDARY OUTCOMES** | | | | | | | |
| Stroke Quality of Life | Survey | SSQoL – stroke specific, health-related quality of life | 27 | 10 | X | X | X |
| Stroke Outcomes | Survey | Rankin | 1 | 1 | X | X | X |
| Physical Functioning | Survey | SF-36V subscale floor effect | 10 | 3 | X | X | X |
| Depression | Survey | PHQ9 | 9 | 3 | X | X | X |
| Self-efficacy | Survey | Self-Efficacy | 9 | 3 | X | X | X |
| Outcome Expectations | Survey | Outcome Expectations | 9 | 3 | X | X | X |
| Knowledge of Stroke Signs | Survey | Warning Signs/Plan for Stroke | 2 | 1 | X | X | X |
| Demographics | Survey | Age, race, income, education, marital status, Mini-Mental Screener | 5 | 1 | X |  |  |
| Stage of Change | Survey | Stage of Change for Physical Activity; Diet, Cigarettes Smoking | 4 | 1 | X | X | X |
| Blood Pressure | CPRS | Systolic/Diastolic | Abstracted  From Medical Record | | X | X | X |
| Body Composition | CPRS | Weight |  |  | X | X | X |
|  | CPRS | Height |  |  | X | X | X |
| Clinical Data | CPRS | Comorbidity; Cholesterol; smoking status; |  |  | X | X | X |
