# Supplementary material for: Using intervention mapping to develop and adapt a secondary stroke prevention program in Veterans Health Administration medical centers
Source: Implement Sci. 2010 Dec 15;5:97. doi: 10.1186/1748-5908-5-97 (PMC3057184; doi:10.1186/1748-5908-5-97)
Supplement: Additional file 2 — Prescription Pad. The additional file includes an example of the 'prescription pad' we used to help management of stroke risk factors for our specific VA hospital. [file 1748-5908-5-97-S2.PDF]

Rx for Stroke Risk Factor Reduction

Stroke Risk Factors that **cannot** be controlled:

Age; Race; Family History; Gender; Previous TIA, Stroke or Heart Attack

Stroke Risk Factors that **can** be controlled:

Stress

- **Primary Care Behavior Specialist ***

or

Roudebush Mental Health
988-XXXX Rm. D1039

Poor Diet

- VA Nutrition Education/ Dietician*

Dietician/ Nutrition Education
988-XXXX Rm. C-7141

Obesity

- VA "Move" Weight Loss Program

Move Weight Loss Program
Kathryn Shanahan Move Coordinator
Call patient response center for Move appt.

Cholesterol

- Cholesterol Clinic* 988-2633 C-3004
- Nutrition Education*

Nutrition Education
988-XXXX Rm. C-7141

Smokers

- VA Smoking Cessation Class (4 sessions/1per week)

&

- 1-800-QUIT-NOW

VA Smoking Cessation Class
988-XXXX Rm. C-1183

Other Resources

Patient Response Center (Clinic Appointments)
988-4498 or 1-888-342-7602
Patient Advocate
988-XXXX

***Items require MD, NP or PA order**

Substance Abuse

- SATS Program (Substance Abuse)

SATS Program/ Substance Abuse
988-XXXX Rm. C1183

Artery & Heart Diseases

- Warfarin/ Coumadin Clinic*

Warfarin/ Coumadin Clinic
988-XXXX Cold Springs Road Bldg. #41

Diabetes

- Diabetes Clinic*
- Diabetes Education Center*

Diabetes/Endocrine Clinic
988-XXXX Rm C-3004
Diabetes Education
988-XXXX Rm A-2015
Dietician/ Nutrition Education
988-XXXX Rm C-7141

Lack of Exercise

- VA Outpatient Rehab OT/PT/KT

OT Outpatient
988-XXXX Rm D-4030

PT Outpatient
988-XXXX Rm D-4030

High Blood Pressure

- Hypertension Clinic*
- Home B/P Monitor Instruction

Hypertension/ Cholesterol Clinic
988-XXXX Rm. C-3004
